# Shotgun metagenomics reveals the prevalence and mobility of antibiotic resistance genes in the West Bay of the human-impacted Laguna Lake

**DOI:** 10.3389/fmicb.2026.1742578

**Published:** 2026-01-22

**Authors:** Lance Matthew F. Farinas, Laurice Beatrice Raphaelle O. dela Peña, Windell L. Rivera

**Affiliations:** Pathogen-Host-Environment Interactions Research Laboratory, Institute of Biology, College of Science, University of the Philippines Diliman, Quezon City, Philippines

**Keywords:** antibiotic resistance genes, antimicrobial resistance, Laguna Lake, mobile genetic elements, One Health, shotgun metagenomics

## Abstract

Laguna Lake, the largest freshwater lake in the Philippines, has been reported to harbor antibiotic-resistant bacteria, posing health risks to the millions who depend on it. However, limited knowledge of antibiotic resistance genes (ARGs) in the lake highlights the need for a comprehensive assessment of its resistome. In line with this, we characterized ARGs in the West Bay of Laguna Lake using shotgun metagenomic sequencing based on six metagenomes collected from three stations across two sampling months at a single depth. ARGs were quantified from short reads, and assembled contigs containing these genes—antibiotic-resistant contigs (ARCs)—were analyzed to assess mobility through associations with plasmids and mobile genetic elements (MGEs). β-lactam resistance genes (0.023–0.048 copies per cell) were the most prevalent, corroborating previous reports. Meanwhile, the detection of bacitracin (0.013–0.028 cpc) and polymyxin (0.009–0.011 cpc) resistance genes raises new concerns, as resistance to these antibiotic classes has not been previously reported in the lake. Furthermore, 44.8 and 30.4% of ARCs were associated with plasmids and MGEs, respectively. ARCs carrying genes for resistance to β-lactams, chloramphenicol, and tetracyclines were frequently identified as mobile, indicating a high potential for horizontal gene transfer and suggesting possible antibiotic contamination in the lake. Overall, this study provides the first metagenomic insight into the resistome of Laguna Lake using short-read sequencing and highlights its role as an environmental reservoir of mobile ARGs. The findings underscore the need for expanded ARG surveillance to improve antimicrobial resistance risk prediction.

## 1 Introduction

Antimicrobial resistance (AMR) is a growing public health concern as drug-resistant strains continue to appear and cause millions of deaths worldwide each year (Zhang A. N. et al., 2021; Parmanik et al., 2022). The widespread use of antibiotics by humans introduced unprecedented selection pressures that sped up the evolution and spread of AMR among microbial populations, overtaking the development of antibiotics used to combat bacterial infections (Edwards et al., 2021; Larsson and Flach, 2022). With the conception of the One Health approach, the role of the environment in the emergence of AMR is given importance. Current research shows that the environment significantly contributes to the rise of antibiotic-resistant bacteria (ARB) by serving as reservoirs for antibiotic resistance genes (ARGs) that can be acquired by previously non-resistant bacteria through horizontal gene transfer (Larsson and Flach, 2022). Hence, environmental monitoring of ARGs to assess AMR threat and inform mitigation policies is a viable strategy to combat the emergence of resistant pathogens.

Freshwater ecosystems are areas of interest for environmental monitoring because they are highly susceptible to contamination from sources such as urban wastewater and agricultural runoff (Nnadozie and Odume, 2019). Contaminants such as antibiotic residues, heavy metals, and organic nutrients can create selection pressures that favor ARBs and lead to the enrichment of ARGs in the ecosystem (Li et al., 2019; Wang et al., 2020; Zhang Y. et al., 2021). Additionally, human and animal wastes can introduce ARBs that evolved endogenously within hosts as a result of widespread antibiotic use in both human and veterinary medicine (Karkman et al., 2019; Czatzkowska et al., 2022; Larsson and Flach, 2022).

Laguna Lake, the largest inland freshwater lake in the Philippines, is currently classified as Class C by the Department of Environment and Natural Resources (DENR), indicating suitability for fisheries, irrigation, and recreational use (DENR, n.d.). The lake is reportedly used for aquaculture, agriculture, recreation, domestic water supply, and as a potable water source (Salvador-Membreve and Rivera, 2021; dela Peña et al., 2022). Under the regular monitoring of the Laguna Lake Development Authority (LLDA), Laguna Lake and its tributaries have been reported with various levels of contamination with heavy metals and fecal coliform (LLDA, 2024). Recent studies also provide evidence of the presence of ARBs and ARGs in Laguna Lake (Ntabugi et al., 2021, 2023; Salvador-Membreve and Rivera, 2021; dela Peña et al., 2022; Castro et al., 2024), subsequently posing health risks to the 16 million people living in its vicinity (dela Peña et al., 2022).

Existing studies on ARGs in Laguna Lake and neighboring bodies of water have primarily utilized PCR-based methods targeting a limited set of genes that confer resistance to sulfonamides, aminoglycosides, β-lactams, and tetracyclines (Suzuki et al., 2013; Vital et al., 2018; Salvador-Membreve and Rivera, 2021; Mamawal et al., 2023). Moreover, current ARG monitoring strategies rely on isolation-based methods using representative species, usually *Escherichia coli* (Salvador-Membreve and Rivera, 2021; Mamawal et al., 2023). The aforementioned strategies, while valued for their accurate estimation of genotypic resistance, often miss many ARGs (Bai et al., 2022; Burch et al., 2022). Thus, the limitations in methods used present a likely underestimated AMR threat within Laguna Lake, calling for a broader surveillance strategy.

With the developments in and increased accessibility to next-generation sequencing (NGS), comprehensive sampling of all genes present in an environmental sample is now possible. Specifically, shotgun metagenomic pipelines combine environmental genetic data with computational techniques to allow taxonomic, functional, and genetic profiling of the microbiome (Quince et al., 2017). And yet, despite evidence of AMR in Laguna Lake and the emergence of metagenomic analyses, evaluations of its overall ARG profile remain limited. Hence, we sought to contribute new insights into the state of AMR in the lake by utilizing short-read shotgun metagenomics to elucidate the diversity, abundance, and mobility of ARGs. The findings can guide future research and the development of monitoring and mitigation strategies within the lake and similar freshwater environments in the Philippines.

## 2 Materials and methods

### 2.1 Study area and sample collection

The study focused on the West Bay, the largest distinct section of Laguna Lake and the area closest to Metro Manila, a major source of urban wastewater. This proximity makes the West Bay a highly relevant study site, as urban centers often contribute to the persistence of ARGs in aquatic environments through high rates of antibiotic use and substantial wastewater discharge (Díaz-Torres et al., 2024).

**Figure 1:**
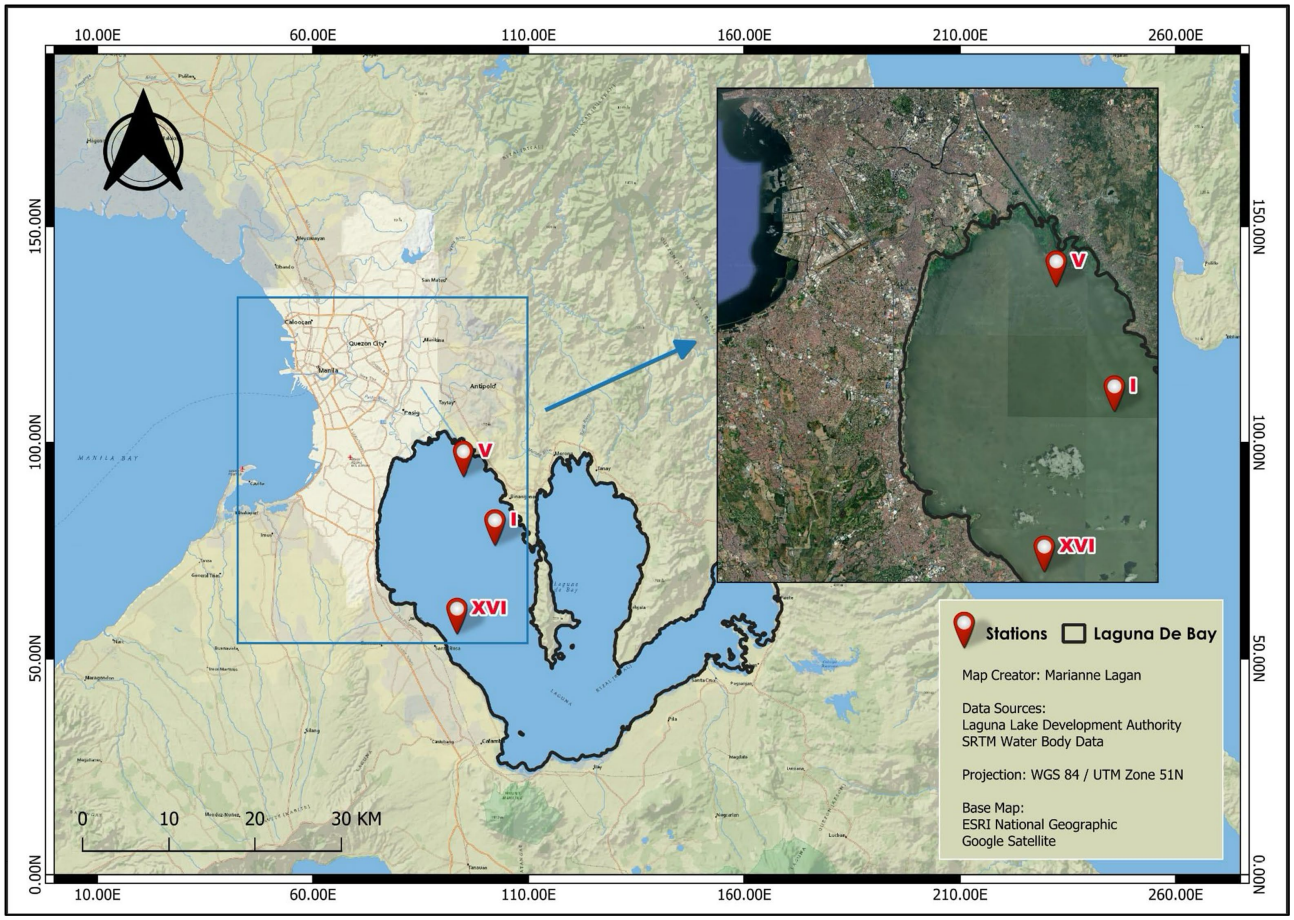
Sampling sites in the West Bay of Laguna Lake selected using data from the Laguna Lake Development Authority (LLDA) lake monitoring stations.

To investigate the ARG profile of the West Bay, three lake stations (LS) were chosen to represent the whole area, namely LS1 (Central West Bay; 14.415556°N, 121.173611°E), LS5 (Northern West Bay; 14.483844°N, 121.134035°E), and LS16 (Sta. Rosa; 14.370446°N, 121.095687°E) as shown in Figure 1. To make the sampling more representative of the West Bay, two water samples were collected from each station during the rainy season, specifically, in August and September 2024. We collected samples in coordination with LLDA’s monthly water quality monitoring. Grab samples from the water surface were temporarily stored in 1 L sterile PET bottles and were transported to the laboratory on ice. The water samples were filtered using 0.45 μm nitrocellulose membranes (Pall Corp., USA) within 24 h of sample collection. Membranes were then placed in DNA/RNA Shield (Zymo Research, USA) and stored at 4 °C until DNA extraction.

### 2.2 DNA extraction and metagenomic shotgun sequencing

Total eDNA was extracted using the Omega E.Z.N.A.® Soil DNA Kit (Omega Bio-tek, USA). Filter membranes were cut into small pieces and transferred into disruptor tubes with the SLX-Mlus Buffer. To remove sediments and lyse cells from the membranes, the tubes were vortexed and then incubated at 70 °C for 10 min with the DS Buffer, followed by another 10 min on a bead beater. The tubes were then centrifuged at 10,000×*g* for 2 min, and the supernatants were transferred into microcentrifuge tubes. Another round of centrifugation was performed to remove any remaining solid particles. After this, the extraction protocol of the kit was followed.

DNA concentrations were assessed using a Qubit Fluorometer. The DNA samples were then temporarily stored at 4 °C before being sent to a commercial sequencing facility for next-generation sequencing (Macrogen, South Korea). Library preparation was performed using the Illumina Nextera XT DNA Kit. Sequencing was conducted on the Illumina NovaSeq platform to generate 2 × 150 bp paired-end reads, which were deposited in the NCBI Sequence Read Archive (SRA) under BioProject accession PRJNA1381765, with run accessions SRR36471021–SRR36471026. Raw reads were then pre-processed for downstream analyses using Fastp v0.24.0 (Chen, 2023) to trim adapter sequences, remove poly-G and poly-X tails, perform paired-end base correction, and filter reads based on quality (Phred >15).

### 2.3 ARG profiling

Antibiotic resistance genes (ARGs) were detected and quantified using ARGs-OAP v3.2.4 (Yin et al., 2023). Annotation and quantification were performed using the default parameters. In ARGs-OAP, ARG types refer to the antibiotics against which the encoded proteins confer resistance, comparable to antibiotic or drug classes used in other studies, while ARG subtypes refer to the specific gene genotypes. In addition, we grouped ARG subtypes into ARG families based on the Comprehensive Antibiotic Resistance Database (CARD) classification (Alcock et al., 2023). The abundance of each ARG type or subtype was calculated as the sum of all reads classified within the same category, normalized to the ARG reference sequence length and the estimated number of prokaryotic cells, as shown in the following equation:


$$\text{Abundance}=\sum_{i=1}^{n}(k\times \frac{Ni_{\text{mapped reads}}\times L_{\text{reads}}/L_{\mathrm{ARG}\;\text{reference sequence}}}{N_{\text{cell}}})$$

where *n* is the number of ARGs belonging to the same category, *Ni*_mapped reads_ is the number of reads mapped to an ARG, *L*_reads_ is the length of the reads, *L*_ARG reference sequence_ is the length of the reference ARG sequence, and *N*_cell_ is the cell number for each sample as calculated in stage one of the ARGs-OAP pipeline. The correction factor *k* was introduced in v3.0 to account for ARG subtypes that utilize two- or three-component systems to encode functional resistance. It is set to 0.5 for ARGs with two-component systems, 0.33 for those with three-component systems, and 1.0 for all other ARGs (Yin et al., 2023).

### 2.4 Assembly of short reads and identification of antibiotic-resistant contigs (ARCs)

To obtain a more comprehensive profile of the ARGs, *de novo* assembly of the clean, short reads into longer contigs was performed using metaSPAdes v3.15.5 on the Galaxy EU platform^1^ using default parameters (Nurk et al., 2017). Each sample was assembled individually.

Antibiotic-resistant contigs (ARCs) were then identified from the assembled contigs following the methods of recently published works (Zhang et al., 2019; Chen et al., 2022). The lengths of the assembled contigs were first determined using SeqKit v2.9.0 (Shen et al., 2024), and only contigs ≥500 bp were retained for downstream analyses (Chen et al., 2022). Open reading frames (ORFs) within contigs were predicted using Prodigal v2.6.3 in meta mode, which is optimized for metagenomic datasets (Hyatt et al., 2010).

The translated ORFs were aligned with the SARG v3.2.1-S database (Yin et al., 2023) using DIAMOND v2.1.11.165 (Buchfink et al., 2021) with the BLASTP command, with cutoffs set at 10^−7^ e-value, 70% identity, and 70% query cover. A contig was considered an ARC if it contained at least one ORF that matched to an ARG (Zhang et al., 2019; Chen et al., 2022).

### 2.5 Characterization of the mobility of ARCs

Plasmid prediction for the identified ARCs was performed using PlasFlow v1.1 (Krawczyk et al., 2018) on the Galaxy EU server. Contigs confidently classified as plasmid-derived are reported as “Plasmid” in this paper, while others are labeled “Non-Plasmid,” indicating potential mobility for those identified as plasmid-like.

All translated ORFs from the identified ARCs were also queried against the mobile orthologous genes (MobileOG) database (v2.0.1–90 [pre-release])^2^ (Brown et al., 2022) using DIAMOND with the same alignment parameters used for ARG detection. ARCs that were identified to harbor mobile genetic element (MGE)-associated genes were classified as mobile ARCs (Zhang et al., 2019; Chen et al., 2022).

### 2.6 Quantification of ARCs

The trimmed short reads from each sample were individually mapped to the contigs using BWA v0.7.19 (Li and Durbin, 2009). The resulting SAM files were processed using SAMtools v1.21 (Danecek et al., 2021) for sorting and coverage estimation. The SAMtools coverage command was used to quantify the number of reads mapped to the contigs and to assess contig quality. The abundance of each ARC was then calculated using the following equation, normalized to cell number:


$$\text{Abundance}=\frac{Ni_{\text{mapped reads}}\times L_{\text{reads}}/L_{\mathrm{ARC}}}{N_{\text{cell}}}$$

where *Ni*_mapped reads_ is the number of reads mapped to an ARC, *L*_reads_ is the length of the short reads, *L*_ARC_ is the length of the ARC, and *N*_cell_ is the estimated cell number for each sample, as determined in stage one of the ARGs-OAP pipeline.

### 2.7 Data analysis

Data analyses were primarily conducted using R v4.4.3. The pheatmap package (Kolde, 2019) was used to visualize the distribution of ARG types across the samples. Stacked bar plots were generated using the ggplot2 package (Wickham, 2009) to visualize relative abundances. The complete analysis workflow and code are documented on GitHub at https://github.com/lmfarinas/laguna-lake-ARG-Analysis.

## 3 Results

### 3.1 Extracted eDNA and pre-processed shotgun metagenomes

The extracted eDNA samples had concentrations ranging from 14.1 to 24.0 ng/μL (Table 1). Illumina paired-end sequencing yielded 47.08 M to 52.74 M reads containing 7.11 G to 7.96 G bases, and 93.82–94.98% of the bases had a Phred quality score ≥ Q20. After Fastp processing, 44.82–50.54 M reads containing 6.69–7.58 G bases passed the quality control, and the Q20 bases increased to 95.40–95.85%. Moreover, 0.009–0.042% of the bases were corrected, and the GC content of the samples ranged from 49.78 to 54.59%.

**Table 1:** Summary of eDNA extraction, metagenomic sequencing, and pre-processing results.

Sequencing and quality metrics	AUGLS01	AUGLS05	AUGLS16	SEPLS01	SEPLS05	SEPLS16
eDNA conc. (ng/μL)	21.6	24.0	20.8	19.4	14.1	19.5
Raw reads	50.340764 M	49.213222 M	51.320168 M	47.084554 M	52.739178 M	51.878252 M
Raw bases	7.601455 G	7.394319 G	7.749345 G	7.109768 G	7.963616 G	7.833616 G
Q20 bases before filtering	94.98%	94.75%	94.43%	93.82%	94.30%	94.38%
Clean reads	48.857514 M	49.213222 M	49.499888 M	44.823608 M	50.541248 M	49.903036 M
Clean bases	7.341573 G	7.394319 G	7.433409 G	6.689432 G	7.577931 G	7.495272 G
Q20 bases after filtering	95.85%	95.79%	95.48%	95.40%	95.61%	95.53%
Corrected bases	0.009%	0.011%	0.015%	0.042%	0.027%	0.019%
GC content	52.98%	51.58%	51.18%	54.59%	51.72%	49.78%

### 3.2 Prevalent ARG types and subtypes

**Figure 2:**
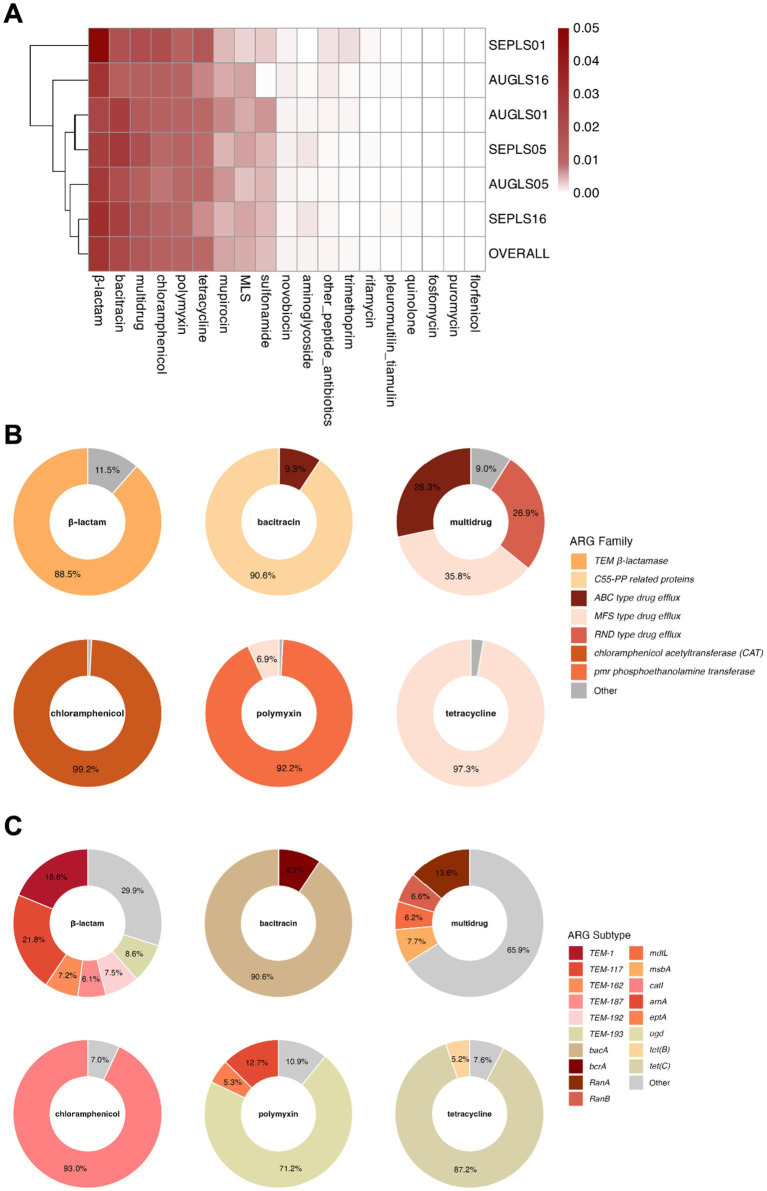
Abundance of ARG types and subtypes in the West Bay of Laguna Lake. **(A)** Heatmap of the abundance (in cpc) of ARG types across the six metagenomic samples. **(B,C)** Relative abundance of ARG families and subtypes, respectively, within the six most prevalent ARG types (overall abundance >0.009 cpc). “OVERALL” represents the cumulative abundance of ARG types from all samples normalized to the total cell copy number. MLS: macrolide-lincosamide-streptogramin. Subtypes or families with less than 5% relative abundance were grouped into “Other.”

A wide range of ARG types was detected across the six water samples (Figure 2A). Based on overall abundance across samples, β-lactam resistance genes were the most abundant overall (0.030 cpc; range across samples: 0.023–0.048 cpc), followed by bacitracin (0.022 cpc; 0.013–0.028 cpc). Multidrug (0.015 cpc; 0.013–0.020 cpc), chloramphenicol (0.011 cpc; 0.008–0.020 cpc), and polymyxin (0.010 cpc; 0.009–0.011 cpc) resistance genes were also consistently detected. Tetracycline (0.009 cpc), mupirocin (0.005 cpc), macrolide-lincosamide-streptogramin (0.005 cpc), and sulfonamide (0.004 cpc) resistance genes were also consistently detected, while the rest were present at negligible levels (<0.001 cpc).

Moreover, analysis at the ARG subtype level showed that a few gene families or specific genes dominated each resistance type (Figures 2B,C). For β-lactam resistance genes, TEM β-lactamases (*bla*_TEM_) accounted for 88.5%. Bacitracin resistance was largely attributed to the undecaprenyl pyrophosphate phosphatase gene *bacA*, which comprised 90.6%. Among multidrug resistance genes, major facilitator superfamily (MFS), ATP-binding cassette (ABC), and resistance-nodulation-cell division (RND) efflux families contributed 35.8, 28.3, and 26.9%, respectively. For chloramphenicol, polymyxin, and tetracycline resistance genes, the most prevalent genes were the chloramphenicol acetyltransferase gene *catI* (93.0%), the UDP-glucose dehydrogenase gene *ugd* (71.2%), and the tetracycline efflux pump gene *tet(C)* (87.2%), respectively.

Complete abundance data for all detected ARG types and subtypes are available in Supplementary Data S1.

### 3.3 Potential mobility of ARCs

**Figure 3:**
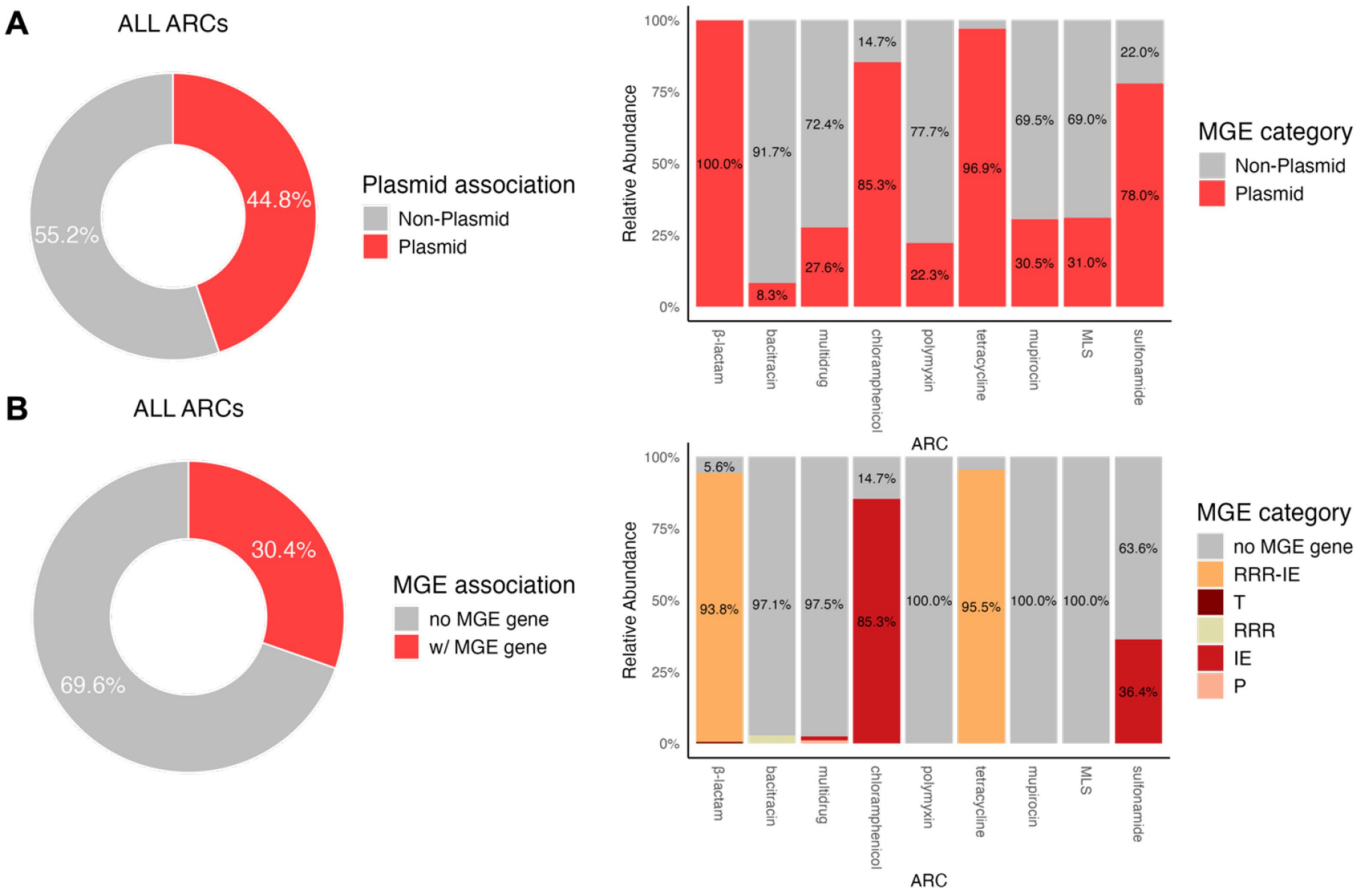
Relative abundance of potentially mobile ARCs with an overall abundance >0.001 cpc. **(A)** Mobility based on plasmid association. **(B)** Mobility based on the presence of MGE-related genes. ARC classification is based on the ARG type they carry. MGE gene types: RRR—replication, recombination, or repair, IE—integration or excision, T—interorganism transfer, P—phage-specific processes.

The potential mobility of ARCs in this study was determined based on whether the contigs were plasmid-associated and whether they contained genes associated with MGEs. As shown in Figure 3A, 44.8% of all ARCs were predicted to be plasmid-associated. Notably, certain ARGs are predominantly carried by plasmid-associated ARCs, including those conferring resistance to β-lactams (100%), chloramphenicol (85.3%), tetracycline (96.9%), and sulfonamides (78.0%). Meanwhile, Figure 3B shows that 30.4% of all ARCs also contain MGEs. ARGs conferring resistance to β-lactams, chloramphenicol, and tetracycline were predominantly found to colocalize with MGE genes. Specifically, β-lactam ARGs were mostly associated with a combination of RRR (replication, recombination, or nucleic acid repair) and IE (integration and excision)-type MGE genes (93.8%). Chloramphenicol resistance genes were primarily associated with IE-type MGEs (85.3%), while tetracycline resistance genes were predominantly colocalized with RRR-IE MGEs (95.5%).

Detailed coverage statistics, abundance profiles (in cpc), ARG and MGE annotations, and plasmid classifications of ARCs are provided in Supplementary Data S2.

## 4 Discussion

This study provides an exploratory snapshot of the resistome of Laguna Lake, and the conclusions should be interpreted in the context of the study’s limited sampling design. The analysis is based on six metagenomes derived from three sampling stations across two sampling months within a single season (wet season), with samples collected at a single depth and without biological replicates. Accordingly, the findings are intended to characterize the diversity, abundance, and mobility of ARGs in the West Bay rather than to assess spatial or temporal variation across the lake. Future studies incorporating expanded spatial coverage, multiple depths, seasonal sampling, and biological replication will be valuable for validating and extending the ARG profiles and mobility patterns observed here.

### 4.1 Genotypic antimicrobial resistance of the lake

We determined the prevalent ARGs in the lake, shedding light on the potential emerging resistance of primary concern. The findings can be used to identify which classes of antibiotics to focus on and which genes to target for environmental monitoring and future research. However, PCR-based quantification of target genes is needed to accurately assess ARG contamination, and antibacterial susceptibility testing is required to confirm phenotypic resistance before formulating monitoring and management policies.

#### 4.1.1 β-Lactam resistance genes

The prevalence of β-lactam resistance genes in the lake is supported by the observed phenotypic resistance in bacterial isolates against β-lactams and the detection of related genes—particularly Extended-Spectrum β-Lactamase (ESBL) genes—in previous studies (Salvador-Membreve and Rivera, 2021; Mamawal et al., 2023). In particular, *bla*_TEM_ was the most prevalent gene family among the ESBLs examined (*bla*_CTX-M_, *bla*_TEM_, and *bla*_SHV_), detected in 47.66% of the 214 *E. coli* isolates from Laguna Lake (Salvador-Membreve and Rivera, 2021).

The abundance of β-lactam resistance genes in the environment may be attributed to the fact that β-lactams are the most widely used class of antibiotics. Resistance to this class is commonly due to the production of β-lactamases, enzymes that hydrolyze and inactivate the β-lactam ring (Bush and Bradford, 2016). A major cause for concern is the presence of ESBL genes (e.g., *bla*_TEM_), generally found in *Enterobacteriaceae*, as these encode enzymes that can inactivate a broad range of β-lactams, including later generations (Castanheira et al., 2021).

#### 4.1.2 Bacitracin resistance genes

Another prevalent ARG-type detected in the present study is bacitracin resistance. This is a novel finding, considering that bacitracin resistance has never been reported in any genotypic or phenotypic studies within Laguna Lake. Bacitracin is primarily used against Gram-positive bacteria; however, since prior studies focused on Gram-negative isolates, bacitracin resistance was neither targeted in PCR detection nor assessed through antibiotic susceptibility testing. Nevertheless, bacitracin resistance genes have been reported in a crab pond wastewater purification system (Chen et al., 2022) and in a non-intensive aquaculture farm (Tian et al., 2024) in China. Moreover, a study examining publicly available metagenomes of freshwater lakes from various countries identified bacitracin as among the most widely detected ARGs globally (Chen et al., 2019). Therefore, while the findings from Laguna Lake are novel at the local scale, they reflect global trends in the spread of environmental antibiotic resistance. This result underscores the importance of including bacitracin resistance in future research in the Philippines to better assess its prevalence and identify contributing factors.

Moreover, the dominant subtype of bacitracin resistance identified in this study was the *bacA* gene. In *E. coli*, *bacA* encodes the undecaprenyl pyrophosphate phosphatase (UppP), which rapidly dephosphorylates undecaprenyl pyrophosphate (C55-PP) to undecaprenyl phosphate (C55-P) (Ghachi et al., 2004). C55-P is a crucial lipid carrier involved in transporting hydrophilic precursors across the cell membrane for the biosynthesis of peptidoglycan, lipopolysaccharide, and teichoic acids. Since bacitracin targets C55-PP, the rapid conversion to C55-P by UppP confers resistance (Ghachi et al., 2004). Additionally, *bacA* homologs in clinically relevant Gram-positive bacteria, such as *Staphylococcus aureus* and *Streptococcus pneumoniae*, have been implicated not only in bacitracin resistance but also in virulence. A study has shown that *bacA* mutants exhibit increased sensitivity to bacitracin and reduced virulence in mouse models (Chalker et al., 2000).

#### 4.1.3 Polymyxin resistance genes

Another novel finding is the detection of polymyxin resistance genes. Although polymyxin is an important drug for treating Gram-negative infections, its resistance has not been investigated in Laguna Lake. Meanwhile, numerous reports have documented the presence of polymyxin resistance in other countries (Chen et al., 2019, 2022; Tian et al., 2024), indicating that such resistance is already a global concern. Its detection in Laguna Lake potentially raises public health concerns, given that polymyxin is considered a last-resort antibiotic for treating multidrug-resistant Gram-negative infections (Mohapatra et al., 2021). While this suggests that untreatable bacterial strains may already be emerging within the lake ecosystem, more definitive proof through the isolation and resistance characterization of pathogenic bacteria is necessary.

In this study, the dominant gene associated with polymyxin resistance was *ugd*, also known as *pmrE*, which encodes UDP-glucose dehydrogenase, an enzyme involved in the biosynthesis of 4-amino-4-deoxy-L-arabinopyranose (L-Ara4N). Gram-negative bacteria utilize cationic molecules such as L-Ara4N to modify the phosphate groups of lipid A, thereby reducing the overall negative charge of the lipopolysaccharide layer. This modification diminishes the binding affinity of cationic antimicrobial peptides like polymyxin, which depend on electrostatic interactions with negatively charged lipid A to exert their antibacterial effects (Chen et al., 2011; Joo et al., 2023). A previous study has shown that *ugd* expression is correlated with polymyxin B resistance in *Klebsiella pneumoniae* (Chen et al., 2011), while *pmrE* genes identified in environmental samples have also been implicated in contributing to polymyxin resistance (Joo et al., 2023).

#### 4.1.4 Other ARGs

Aside from β-lactam resistance, multidrug and chloramphenicol resistance have also been documented in Laguna Lake and its surrounding waters (Vital et al., 2018; Ntabugi et al., 2021). Previous studies have shown the resistance of *E. coli* isolates through antibacterial susceptibility testing. The present study provides genetic confirmation of these previous findings by reporting the presence of multidrug and chloramphenicol resistance genes in the lake. Quantification of ARGs further showed that these two classes were relatively prevalent (>0.01 cpc) in the West Bay of Laguna Lake. In contrast, sulfonamide, aminoglycoside, and tetracycline resistance genes, which have been previously monitored through PCR-based techniques in Laguna Lake (Suzuki et al., 2013; Vital et al., 2018; Salvador-Membreve and Rivera, 2021), were comparatively less abundant (less than 0.01 cpc) in the current study. Moreover, while prior monitoring efforts in the lake focused solely on the *tet(A)* gene for tetracycline resistance (Salvador-Membreve and Rivera, 2021), we identified *tet(C)* as the dominant tetracycline resistance gene. Together, these findings underscore the value of metagenomic approaches in ARG monitoring, as they enable the identification of dominant resistance gene types prior to the implementation of more extensive surveillance strategies.

The dominant genes associated with multidrug resistance in this study were identified as members of three major efflux pump families: RND, MFS, and ABC transporters. These drug efflux pumps contribute to antibiotic resistance by actively transporting antimicrobial agents out of the cell, thereby lowering intracellular drug concentrations (Yamasaki et al., 2023). In addition to their role in resistance, they are also implicated in various virulence-related processes, including biofilm formation, quorum sensing, and the excretion of potentially harmful host-derived metabolites (Yamasaki et al., 2023). In particular, RND transporters are primarily found in Gram-negative bacteria and typically form a tripartite complex that spans both the inner and outer membranes, facilitating the direct export of substances out of the cell. MFS pumps, on the other hand, are widespread across all domains of life and typically function as single-membrane transporters. Although highly diverse in structure and substrate range, individual MFS pumps are often substrate-specific, including specificity for certain antibiotics. Finally, ABC transporters function as ATP-hydrolyzing, unidirectional pumps that mediate the outward transport of substrates (Zack et al., 2024).

For chloramphenicol resistance, the dominant gene identified was *catI*, which encodes the enzyme chloramphenicol acetyltransferase. This enzyme inactivates chloramphenicol by transferring an acetyl group from acetyl-CoA to the antibiotic molecule. The resulting acetylated form of chloramphenicol can no longer bind effectively to the 50S subunit of the bacterial ribosome, thereby preventing it from inhibiting protein synthesis (Mosa et al., 2015).

### 4.2 Mobile ARGs in Laguna Lake

Plasmids, as mobile genetic elements, enable the rapid dissemination of resistance genes between bacteria (Wang et al., 2023). Notably, conjugative plasmids exhibit significantly higher transfer rates than chromosomal elements. Furthermore, many bacterial species rely on interspecies plasmid transfer as their main mechanism for acquiring ARGs (Lehtinen et al., 2021). In addition to plasmids, other MGEs include transposons, integrons, and bacteriophages, all of which contribute to the dissemination of ARGs via horizontal gene transfer (Brown et al., 2022). In this study, we assessed the potential mobility of ARCs by determining whether the contigs were plasmid-associated and carried MGE genes. Assessing the mobility of ARGs improves risk prediction because if genes are mobile, they can more easily spread across bacterial populations especially to pathogenic species (Lehtinen et al., 2021; Czatzkowska et al., 2022; Wang et al., 2023).

β-lactamase genes, particularly *bla*_TEM_, have been predominantly found on plasmids in clinical *E. coli* isolates from Luzon (Cruz and Hedreyda, 2017). This aligns with the present findings, in which *bla*_TEM_ was the most prevalent β-lactam ARG and all ARCs carrying β-lactam resistance genes were predicted to be plasmid-associated. Moreover, *bla*_TEM_—along with *bla*_SHV_—were among the first plasmid-borne enzymes from which many ESBLs were later derived (Cruz and Hedreyda, 2017; Castanheira et al., 2021). Plasmid-borne *bla*_TEM_ genes have also been frequently associated with transposons and insertion sequences, which are small transposable elements (Castanheira et al., 2021). This supports the present finding that β-lactam ARCs also contain RRR- and IE-type MGE genes, which, according to the mobileOG database classification, are involved in the mobilization processes of plasmids, transposons, and insertion sequences (Brown et al., 2022).

The plasmid association of chloramphenicol ARGs is also supported by previous studies. Chloramphenicol resistance, often conferred by chloramphenicol acetyltransferase (CAT) genes, can be either chromosomal or plasmid-borne (Biswas et al., 2012). CAT genes have also been reported to associate frequently with other MGEs, such as transposons and gene cassettes (Roberts and Schwarz, 2009). In this study, *catI*—the most prevalent chloramphenicol ARG detected—is known to be widespread among Gram-negative bacteria and has been reported to be transposon-borne and commonly located on large plasmids alongside other ARGs (Roberts and Schwarz, 2009). Additionally, *catI* was identified as a plasmid-encoded ARG in multidrug-resistant *Klebsiella pneumoniae* isolates in a study conducted in China (Chen et al., 2023). The present finding that *catI*-carrying ARCs frequently contain IE-type MGEs thus supports its described mobility, consistent with the cited literature.

Tetracycline resistance is commonly acquired through the uptake of genes carried by mobile plasmids, particularly those encoding tetracycline efflux pumps (Roberts and Schwarz, 2009). In this study, *tet(C)* was the primary tetracycline ARG identified. This gene is typically associated with Gram-negative genera and is frequently linked to mobile genetic elements, including plasmids and transposons (Roberts and Schwarz, 2009; Shi et al., 2021). Moreover, *tet(C)* is considered one of the most widely distributed tetracycline resistance genes in environmental settings such as municipal wastewater treatment plants, fishponds, rivers, and soils (Shi et al., 2021). Similar to previously discussed mobile ARCs, the *tet(C)*-carrying ARCs in this study were also found to contain RRR- and IE-type MGE genes, supporting the described mobility of the *tet(C)* gene as reported in the cited literature.

Lastly, sulfonamide resistance is frequently mediated by the acquisition of *sul* genes. In this study, *sul1* and *sul2* were the predominant sulfonamide ARGs associated with ARCs. These genes are commonly plasmid-borne and have been widely reported in clinical isolates of Gram-negative bacteria such as *E. coli*, *K. pneumoniae*, and *Acinetobacter baumannii* (Lin et al., 2021; Venkatesan et al., 2023).

#### 4.2.1 Implications of ARG mobility for antibiotic contamination in Laguna Lake

The prevalence of mobile ARGs in Laguna Lake may imply the presence of antibiotic contamination. In previous literature, the increase in ARGs on conjugative plasmids has been correlated with the growing use of antibiotics worldwide (Wang et al., 2023). Moreover, it has been noted that mobile genes, particularly those that are only moderately beneficial under normal conditions, are especially sensitive to selection pressures. For instance, essential genes are typically located on the chromosome rather than on plasmids due to the risk of plasmid loss during cell division. In the absence of strong selection, plasmid-borne genes offer no advantage and are often lost (Lehtinen et al., 2021). However, under intense selective pressure—such as the presence of antibiotics—bacteria harboring resistance genes gain a fitness advantage. If these genes are mobile, they can be horizontally transferred, facilitating their spread and enrichment in the environment (Lehtinen et al., 2021; Czatzkowska et al., 2022; Wang et al., 2023).

Although there are no published reports confirming the presence of antimicrobial residues in the waters of Laguna Lake, their presence cannot be ruled out, as indirect evidence for antibiotics in the lake suggests their likely presence. For example, β-lactams are among the most commonly misused drugs, according to a 2015 cross-sectional survey conducted in Central Visayas, Philippines (Barber et al., 2017). Meanwhile, tetracycline and chloramphenicol are commonly associated with aquaculture (Tendencia and de la Peña, 2001; Revilleza et al., 2021). Lastly, sulfonamides, which are among the oldest classes of antibacterial drugs, remain widely used in both human and veterinary medicine due to their broad-spectrum activity (Venkatesan et al., 2023). For example, sulfamethoxazole, a sulfonamide, is commonly used in pig farming in the Philippines (Calayag et al., 2021). These suggest potential sources of antibiotic contamination for the lake.

Overall, the prevalence and the mobility of the aforementioned ARGs indicate potential antibiotic contamination in Laguna Lake. The findings underscore the need to expand ARG surveillance in Laguna Lake, to improve AMR risk prediction and to inform evidence-based interventions. Laguna Lake, being situated at the interface of human settlements, agricultural activities, and aquatic ecosystems, represents a critical One Health convergence point where AMR determinants can emerge and disseminate across environmental, human, and animal reservoirs. The present study can thus be viewed as a baseline assessment that establishes the initial reference data on ARG presence in the lake system. Building on this foundation, future studies can integrate longitudinal sampling, clinical and veterinary AMR data, and environmental drivers to integrate surveillance data into actionable management policies.

## Data Availability

Sequencing was conducted on the Illumina NovaSeq platform to generate 2 × 150 bp paired-end reads, which were deposited in the NCBI Sequence Read Archive (SRA) under BioProject accession PRJNA1381765, with run accessions SRR36471021–SRR36471026.

## Footnotes

1
https://usegalaxy.eu/

2
https://zenodo.org/records/14725479

## Supplementary material

Supplementary Data S1 Abundance data of detected antibiotic resistance gene (ARG) types and subtypes in ARGs-OAP. Sheet 1 (ARG_Types): Abundance of ARG types, normalized to copies per cell (cpc), across samples. Sheet 2 (ARG_Subtypes): Abundance of ARG subtypes, normalized to cpc, across samples. “OVERALL” represents the cumulative abundance of ARG types from all samples, normalized to the total cell copy number.

Supplementary Data S2 Detailed information on antibiotic resistance contigs (ARCs). Sheet 1 (ARC_Coverage_Stats): Coverage statistics of ARCs. Sheet 2 (ARC_Profile_Summary): Abundance (copies per cell, cpc), detected antibiotic resistance genes (ARGs), mobile genetic elements (MGEs), and PlasFlow classification of ARCs. Sheet 3 (ARG&MGE_hits): Annotation results from reference databases, including ARG types and subtypes and MGE categories. Sheet 4 (plasmid_ARCs): Relative abundance of ARCs classified as plasmids. Sheet 5 (MGE_ARCs): Relative abundance of ARCs carrying MGEs.
